# Case Report: Unveiling CHARGE syndrome: a neonatal case study with esophageal atresia and feeding difficulties

**DOI:** 10.3389/fped.2025.1618512

**Published:** 2025-10-23

**Authors:** Fangjian Gao, Shuyan Li, Li Hu, Yali Zeng, Jianwu Qiu

**Affiliations:** ^1^ Guangdong Medical University, Zhanjiang, China; ^2^ Department of Neonatology, Yuebei People’s Hospital, Guangdong Medical University, Shaoguan, China; ^3^ Guangdong Province Critical Newborn Rescue Center (North Guangdong), Shaoguan, China

**Keywords:** CHARGE syndrome, CHD7, esophageal atresia, feeding difficulties, congenital disorders

## Abstract

CHARGE syndrome is a collection of congenital malformations resulting from pathogenic variants that cause loss of function in the CHD7 gene. These malformations are characterized by coloboma, heart defects, atresia of the choanae, growth retardation, genital abnormalities, and ear abnormalities. We report a case of neonatal CHARGE syndrome, which presented with congenital esophageal atresia and feeding difficulties. Genetic analysis confirmed the presence of a mutation in the CHD7 gene. The diagnosis of CHARGE syndrome should be considered and confirmed through CHD7 gene sequencing in fetuses with esophageal atresia suspected by prenatal ultrasound, especially when combined with multiple malformations and feeding difficulties post-birth.

## 1 Introduction

CHARGE syndrome(CS)(OMIM #214800) is a collection of congenital malformations characterized by coloboma, choanal atresia, cranial nerve impairment, inner or outer ear malformations, hearing loss, cardiac malformations, genitourinary abnormalities, retardation (of growth and/or development). The majority of individuals are caused by loss-of-function pathogenic variants in the CHD7 gene, with an incidence of approximately 1 in 10,000 births. The condition does not have sex-linked expression, so males and females are equally affected. Most cases are sporadic and are not inherited from their parents (1–3).

We present a case of CHARGE syndrome in a newborn with esophageal atresia and feeding difficulties as the main manifestations, which was confirmed by genetic analysis demonstrating CHD7 mutation. There are relatively few case reports of this phenotype (4). The aim of this report is to enhance the understanding of this syndrome, facilitate early diagnosis, and reduce misdiagnosis.

## 2 Case history

A female infant was admitted to the hospital with postnatal shortness of breath that had persisted for over 2 h, occurring 3 h after birth. She was delivered via spontaneous vaginal delivery at a gestational age of 36 weeks and 6 days, weighing 2,400 g in the obstetrics department of another hospital, with premature rupture of membranes at 13 h. At the time of delivery, the umbilical cord was not encircled, the amniotic fluid volume was appropriate and clear, and the placenta appeared normal. The infant gradually developed shortness of breath after birth, exhibiting mild three-concave sign with froth at the mouth and no cyanosis. She was transferred from a local hospital to our department under low-flow oxygenation by ambulance. At 24 weeks of gestation, prenatal ultrasound revealed polyhydramnios and an absent fetal stomach bubble, and a “oesophageal pouch sign” was seen in the neck, which was considered a possible indication of fetal esophageal atresia. An anomalous right subclavian artery and coalesced echogenic foci of the ventricle were also found.

**Figure 1:**
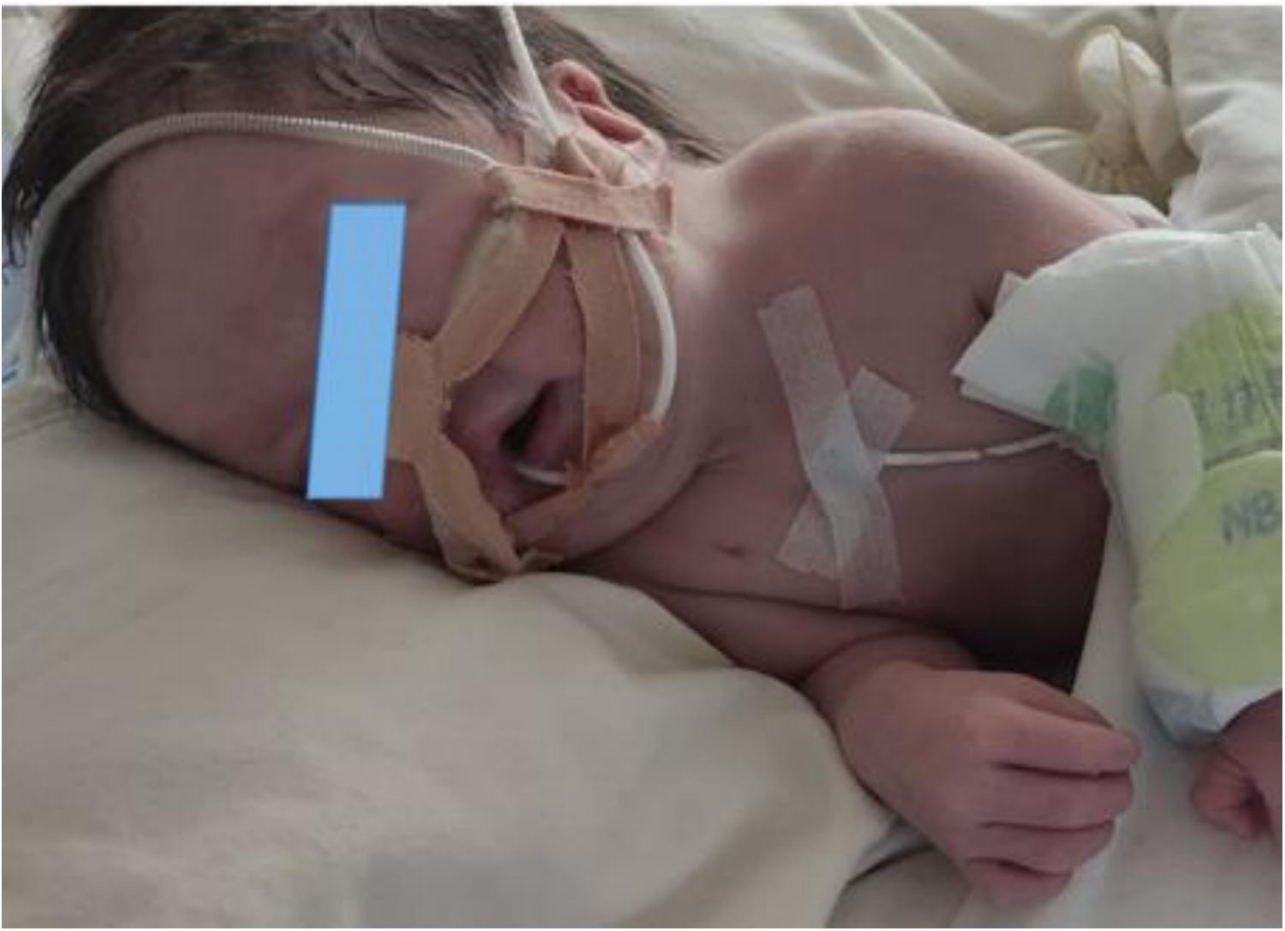
The patient presented with a low-set ears, bossing forehead, short philtrum, short neck and high arched palate.

The physical examination revealed a temperature of T36.6°C, a pulse rate of 145 beats per minute, a respiratory rate of 61 breaths per minute, and blood pressure of 72/41 mmHg. There was no evidence of jaundice, cyanosis, or mottling of the skin and mucous membranes. The patient exhibited some dysmorphic features, including low-set ears, a prominent forehead, a short philtrum, a short neck, and a high-arched palate (Figure 1). The anterior fontanel was flat and soft, and both pupils were equally large and round, with a brisk light reflex. Inspiratory three-concave signs were positive. The patient was tachypneic with coarse breath sounds in both lungs, and wet rales were audible in both lungs. The heart rate was 145 beats per minute with a grade 2/6 systolic murmur heard between the 2nd and 3rd intercostal spaces at the left sternal edge. The abdomen was slightly distended, soft, and normal bowel sounds were present. The liver extended approximately 2 cm below the costal margin with a soft edge. Muscle tone was slightly increased, with incomplete neonatal reflexes.

**Figure 2:**
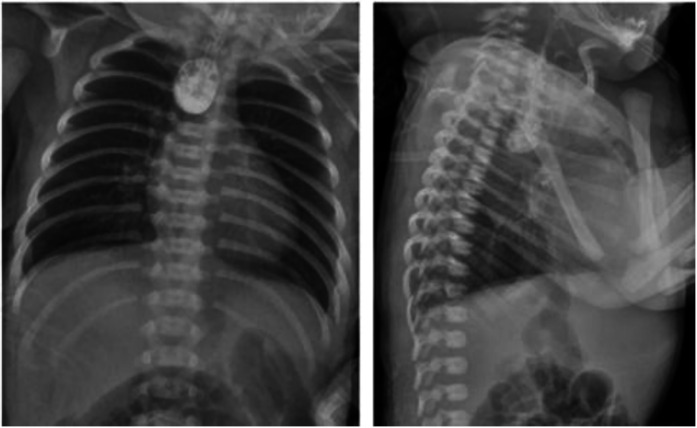
Upper gastrointestinal examination. An appropriate amount of contrast agent was injected through the gastic tube. The upper thoracic segment of the esophagus appeared as a cystic bag shadow, with its lower edge approximately at the level of the lower edge of the third thoracic vertebra. The middle and lower thoracic segments of the esophagus were not visualized.

**Figure 3:**
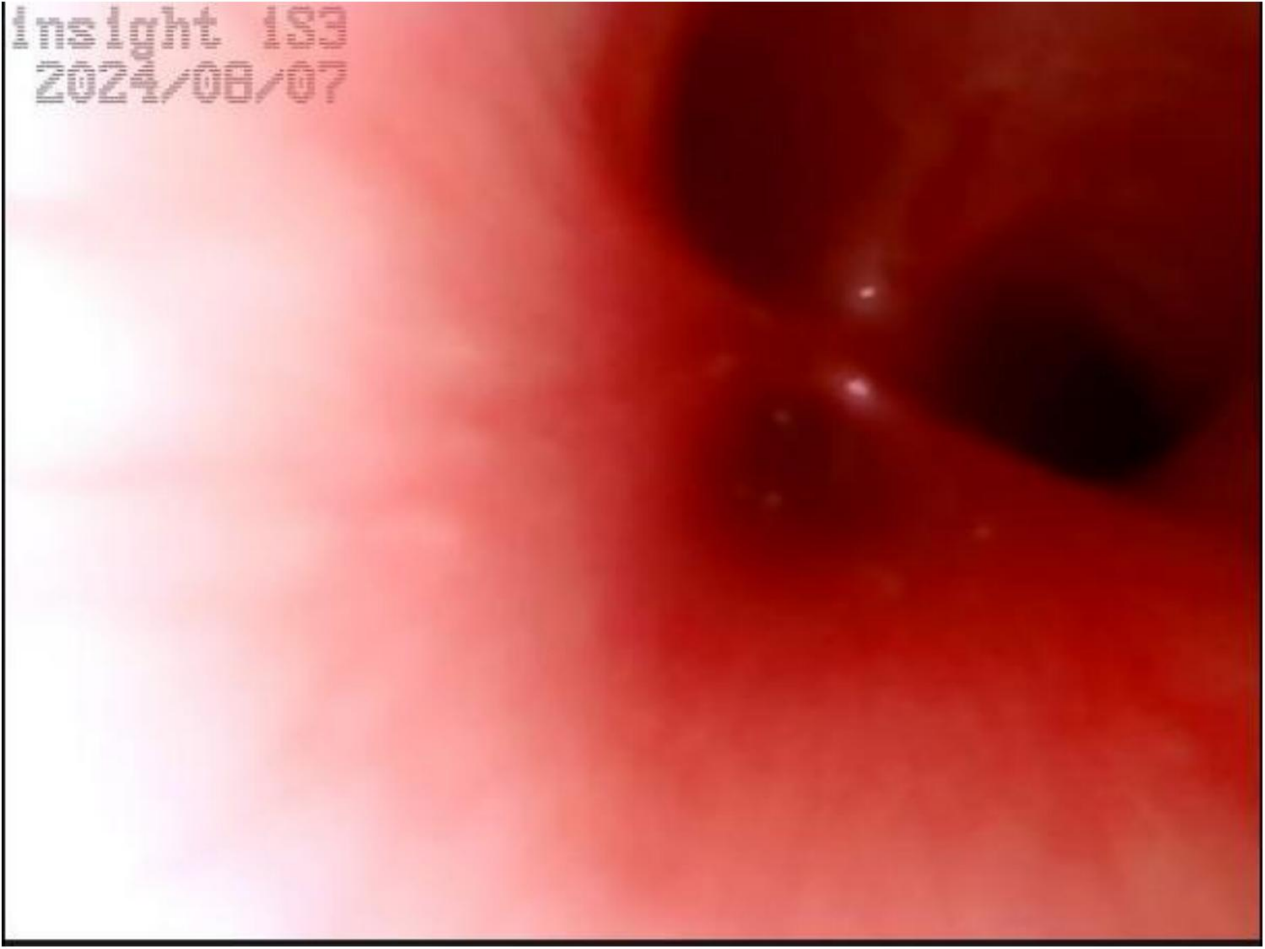
Electron bronchoscopy. At the level of the tracheal carina, an opening of a fistula is visible, with a diameter smaller than that of the left and right main bronchi. There is no swelling, and a small amount of white secretions can be observed.

**Figure 4:**
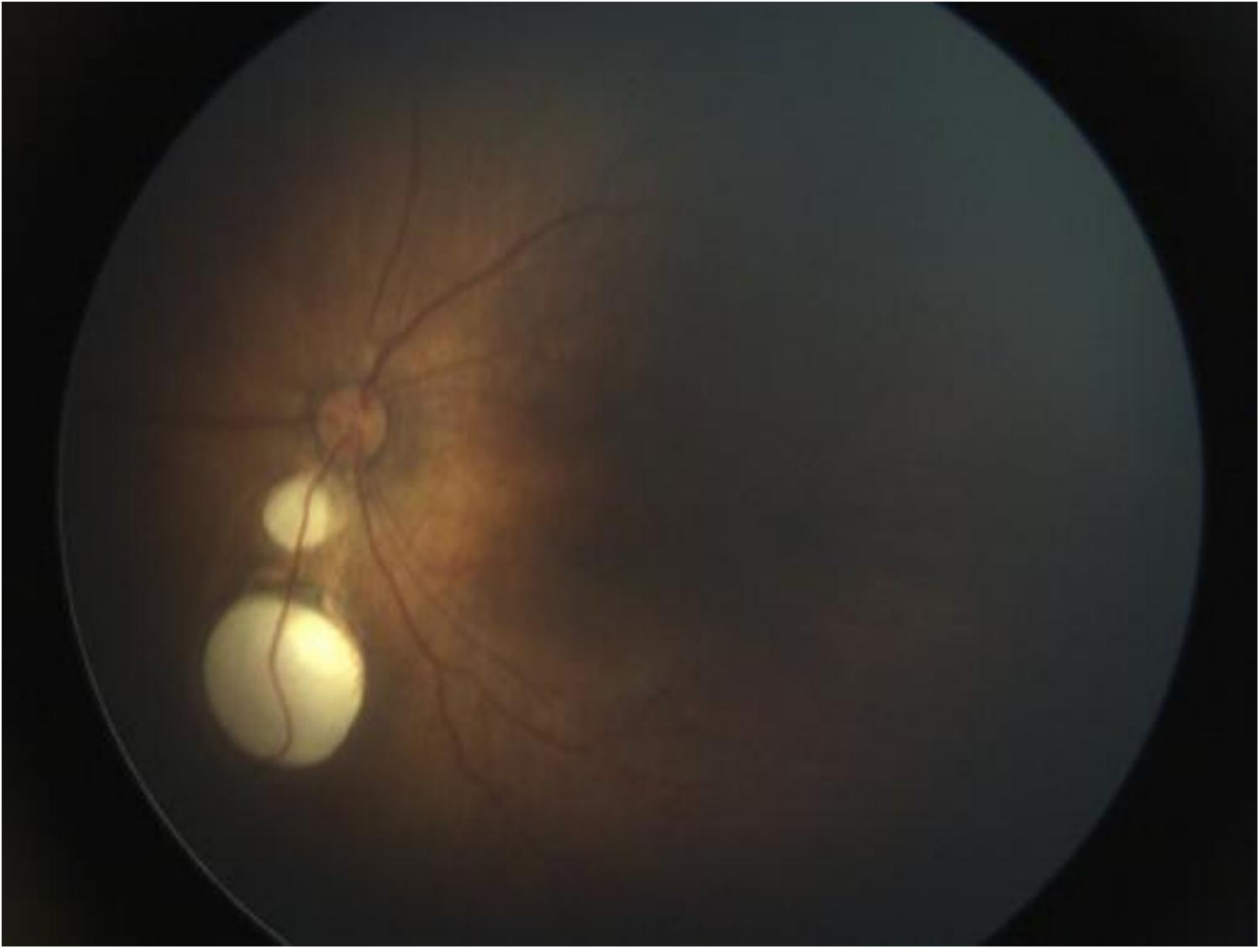
Fundoscopic examination. Focal retinal choroidal defects were seen below the optic disc, no obvious retinal detachment, no fibrous tissue proliferation or neovascularization.

Upper gastrointestinal examination with iodinated water contrast revealed a cystic mass shadow at the level of the lower margin of the third thoracic vertebra (Figure 2). Congenital esophageal atresia (Type C) was suspected based on CT imaging of esophagus. Electron bronchoscopy visualized congenital esophageal atresia with a tracheoesophageal fistula (Figure 3). Craniocerebral ultrasound detected a subventricular cystic lesion on the left side and bilateral dilation of the lateral ventricles. Abdominal color Doppler ultrasound indicated hepatomegaly, with no apparent abnormalities in the gallbladder, pancreas, spleen, or kidneys. Cardiac ultrasound identified an atrial septal defect, patent ductus arteriosus, and patent foramen ovale. Amplitude EEG demonstrated immature sleep-wake cycles (SWCs). Hearing screening using Otoacoustic emissions and the auditory brainstem indicated abnormal results. Fundoscopic examination revealed congenital focal retinal choroidal defects in both eyes (Figure 4). Genetic testing with whole-exome sequencing was positive and identified a *de novo* heterozygous pathogenic variant in the CHD7 gene, confirmed by Sanger sequencing, with both parents being wild type at this locus (Supplementary Figures S1).

Upon admission to our hospital, the infant was placed under general anesthesia and underwent a complex surgical procedure in the operating room: “Intrathoracic Esophagogastric Anastomosis, Esophagotracheal Fistula Repair, and Closed Thoracic Drainage,” with endotracheal intubation. Post-surgery, the patient received comprehensive care in the Neonatal Intensive Care Unit (NICU), including ventilator support, anti-infection therapy, nutritional support, and various other supportive treatments. During the treatment period, the infant experienced recurrent apnea, challenges in weaning off oxygen therapy, feeding difficulties, gastroesophageal reflux, and laryngeal stridor following the removal of the ventilator. With aggressive treatment, the infant's condition gradually improved, and she was discharged on the 50th day of hospitalization. After discharge, the infant may exhibit irritability and a slight decrease in oxygen saturation during breastfeeding, which typically returns to normal once feeding is completed and the infant is calmed.

## 3 Discussion and conclusion

Esophageal atresia (EA) is a rare congenital disorder with an incidence of 2.3 cases per 10,000 live births (4). It has five different anatomical subtypes (A through E), and in most cases of EA, there is a tracheoesophageal fistula (TEF). Subtype C of EA is the most common, characterized by the upper segment of the esophagus being closed and the distal segment being connected to the trachea through a fistulous tract (5). Over half of patients with EA have associated birth defects or other abnormalities. Approximately 10% of patients with non-random VACTERL (Vertebral defects, Anal atresia, Congenital heart disease, TEF, Renal anomalies, and Limb abnormalities) association. Additionally, 6% of patients with Trisomy 18 (Edwards syndrome), and 1%–3% with Trisomy 21 (Down syndrome). Furthermore, 1% of patients with EA also have CHARGE (coloboma, heart defects, atresia choanae, growth retardation, genital abnormalities, and ear abnormalities) syndrome (6, 7).

CHARGE syndrome was first described by American dysmorphologist Bryan Hall in 1979, who identified the condition in 17 children with multiple congenital abnormalities (8). Concurrently, ophthalmologist Helen Hittner also described 10 children with similar characteristics, and hence it was initially called Hall-Hittner syndrome (9). In 1981, Pagon et al (10) further ascertained and reported on additional children with choanal atresia or clefts and related characteristic malformations, and first coined the acronym CHARGE association (Coloboma, Heart Defect, Atresia Choanae, Retarded Growth and Development, Genital Hypoplasia, Ear Anomalies/Deafness). CHARGE syndrome was previously referred to as the CHARGE association until 2004, when the chromodomain helicase DNA-binding protein 7 gene (CHD7, OMIM #214800), located on chromosome 8q12.1, was identified as the causative gene responsible for the syndrome, leading to the replacement of the association with the CHARGE syndrome (3, 11).

The chromodomain helicase DNA-binding (CHD)protein family is a group of highly conserved nuclear proteins, with cellular functions of CHD proteins being very diverse, but their role in transcriptional regulation is emerging as a common theme. There are 9 members of CHD proteins in vertebrates, among which CHD7 is of particular interest. The CHD7 gene controls the production of a protein that regulates developmental pathways through chromatin organization. Mutations in the CHD7 gene lead to the production of abnormal, non-functional short CHD7 proteins, which result in increased DNA methylation of the rRNA promoter, thereby reducing rRNA expression. This disruption in the regulation of gene expression and disordered neural crest development can occur. Neural crest cells play a crucial role in giving rise to its derivatives, including bone, pigment, smooth muscle, endocrine cells, neurons, glial cells, and cartilage. Insufficient migration of cervical neural crest cells into the pharyngeal pouches and arches, lack of mesodermal development, and defective interface between neural crest cells and mesoderm can subsequently lead to a variety of phenotypic abnormalities. If these changes occur during embryogenesis, it leads to the development of the typical pleiotropic signs and symptoms of CHARGE syndrome (12–14). Mutations in the CHD7 gene are the most common cause of CHARGE syndrome, which is inherited in an autosomal dominant pattern, with the majority of cases being caused by *de novo* mutations, meaning that they are not inherited from their parents, but occur sporadically (2). The infant in this case has a heterozygous variation c.3205C>T (Arg1069*) in the CHD7 gene, with both parents being wild type. This variation site is located in the “SNF2 family N-terminal domain and the DEAD-like helicase superfamily and helicase ATP binding” domain, and this variation has been reported in previous literature (15). Unlike the cases described by Lalani et al. (15), the presence of esophageal atresia in our patient represents a phenotypic expansion for the genotype (Arg1069*).Upon querying the web-based database (https://www.chd7.org/), which gives an up-to-date overview of all the described CHD7 mutations and clinical phenotype of patients, where this variation is defined as a Pathogenic nonsense mutation and predicted to result in a loss of function.

CHARGE syndrome has considerable phenotypic variability, and many of its features (including genital hypoplasia, cleft palate, and cardiac defects) are shared with those of other syndromes, such as the Kallmann, VACTERL association. In addition, some clinical features may not be fully expressed early in life, and the differential diagnosis may be challenging for the neonatologist (16). Genetic testing is beneficial to the diagnosis and differential diagnosis of CHARGE syndrome. Clinical features of individuals with CHARGE syndrome who have a confirmed pathogenic CHD7 variant include (3): external ear anomaly (95%), semicircular canal anomaly (94%), coloboma (80%), choanal atresia (49%), Cleft lip and/or palate (40%), cranial nerve dysfunction (94%), feeding difficulties (82%), facial palsy (58%), anosmia (80%), genital hypoplasia (68%), congenital heart defects (78%), tracheo-esophageal anomaly (25%), developmental delay (99%), intellectual disability (79%), and growth retardation (55%).

Blake et al. (17) proposed diagnostic criteria in 1998, which were further modified and revised by Verloes (18). In 2016, Hale (19) proposed new diagnostic criteria for CHARGE syndrome, consisting of 4 major criteria and 7 minor criteria. The major criteria include coloboma, choanal atresia or cleft palate, abnormal external, middle or inner ears, and pathogenic variants in the CHD7 gene. The minor criteria include: ① Cranial nerve dysfunction (including hearing impairment); ② Dysphagia or feeding difficulties; ③ Abnormal brain structure; ④ Developmental delay/autism or intellectual disability; ⑤ Hypothalamic-pituitary dysfunction (deficiency in sex hormones/growth hormone) and gonadal abnormalities; ⑥ Cardiac or esophageal malformations; ⑦ Renal anomalies, skeletal or digital abnormalities. The inclusion criteria for CHARGE syndrome are two major criteria plus any number of minor criteria. Genetic testing is becoming increasingly important in the diagnostic process of CHARGE syndrome. In this case, the infant exhibits three major criteria: coloboma, external ear abnormalities (Low ear), and pathogenic variants in the CHD7 gene. Additionally, the infant presents with four minor criteria: hearing loss, feeding difficulties, brain structural abnormalities (ventriculomegaly, widened interhemispheric fissure), and malformations of the heart (atrial septal defect, patent ductus arteriosus, patent foramen ovale) and esophagus (congenital esophageal atresia type C). The diagnosis of CHARGE syndrome is definitive. (Comparison of Diagnostic Criteria for CHARGE Syndrome, see Supplementary Table S1).

The patient in this case is a premature infant born at 36 weeks and 6 days. She presented with polyhydramnios and an absent fetal stomach bubble, as observed by antenatal ultrasonography at 24 weeks. An anomalous right subclavian artery and coalesced echogenic foci in the ventricle were also detected. The diagnosis of esophageal atresia (Type C) was confirmed through surgery. Post-surgery, the patient experienced recurrent apnea, difficulty weaning off oxygen treatment, feeding difficulties, gastroesophageal reflux, and laryngeal stridor. She exhibited several dysmorphic features, including low-set ears, a bossing forehead, a short philtrum, a short neck, and a high-arched palate. Additionally, she had coloboma in both eyes, hepatomegaly, and congenital heart defects, such as patent ductus arteriosus, patent foramen ovale, and an atrial septal defect. Cranial ultrasound revealed widening of the bilateral ventricles and the longitudinal fissure of brain, while amplitude EEG showed immature sleep-wake cycles (SWCs). The infant failed the hearing screening for both ears. Finally, CHD7 gene sequencing was performed to confirm the diagnosis of CHARGE syndrome. Sucking difficulties are present in almost all neonates with CHARGE syndrome (CS), with approximately half exhibiting breathing-swallowing discoordination (20). Daily feeding in these infants involves a complex interplay of craniofacial anomalies, structural defects, neurological impairment, and prematurity. The postoperative challenges observed in our patient—recurrent apnea, difficulty weaning off oxygen treatment, gastroesophageal reflux, and laryngeal stridor—align with the cranial nerve dysfunction described by Squires LA et al. (21), which leads to impaired swallowing coordination and respiratory abnormalities, explaining the extreme difficulty in managing these infants postoperatively. In contrast to the case reported by Yokota N et al. (22), where severe congenital heart disease was not evident in the neonatal period, our patient presented with patent ductus arteriosus and atrial septal defect requiring medical intervention. This underscores the variability of cardiovascular involvement in CHARGE syndrome and its significant impact on early clinical management. The majority of malformations associated with CHARGE syndrome are difficult to diagnose prenatally by ultrasound, which can only detect a subset of anomalies such as orofacial clefts, and cardiac, renal, and limb defects (23); however, these findings are non-specific. Nevertheless, prenatal diagnosis is crucial due to the poor prognosis associated with CHARGE syndrome. Compared to previous literature, our case presented a combination of highly suggestive prenatal features. The prenatal findings of polyhydramnios and an absent stomach bubble in our case were accompanied by clues to cardiac and vascular anomalies, namely an aberrant right subclavian artery and cardiac echogenic foci, which have been recognized as potential prenatal indicators of CHARGE association in the review by Busa T et al. (23). Certainly, fetal MRI examination can also detect the presence or absence of malformations such as eyes, posterior nares, and internal/external ears, further assisting in diagnosis (24). Esophageal and tracheal malformations, as minor manifestations of CHARGE syndrome, occur in only 18%∼29% of CHARGE syndrome cases (15, 25), which makes it easy for clinicians to overlook the diagnosis of CHARGE syndrome. Suspected cases should undergo prompt genetic sequencing to facilitate genetic counseling and enable precise management based on a confirmed diagnosis (26). In this case, polyhydramnios and an absent gastric bubble were detected prenatally, raising suspicion for esophageal atresia. Postnatal evaluation revealed concomitant congenital heart disease and feeding difficulties, which led to clinical suspicion of CHARGE syndrome. The diagnosis was subsequently confirmed through genetic testing (24, 27).

CHARGE syndrome is currently an incurable congenital disorder. Its management relies on symptomatic and supportive treatment through multidisciplinary collaboration to improve patient survival and quality of life. The case presented in this study, together with previous reports (21, 22, 28–36) (see Supplementary Table S2), collectively reveals significant common features in such patients, particularly when associated with EA/TEF: Gross type C EA/TEF is the most common, and it is frequently combined with congenital heart defects, coloboma, hearing loss, and severe feeding difficulties due to cranial nerve dysfunction. These characteristics highlight that the focus of early intervention should be on maintaining respiratory stability, surgically correcting cardiac and esophageal malformations, and actively addressing feeding challenges.Notably, the detailed prenatal clues presented in this case (such as an aberrant right subclavian artery and cardiac echogenic foci), the factor of prematurity, and severe postoperative breathing-swallowing discoordination further deepen our understanding of the complexity of this condition. It strongly confirms that feeding disorders are by no means solely due to anatomical abnormalities but are also closely related to neurological and sensory deficits (21). This explains why, even after technically successful surgery, patients generally require long-term tube feeding and intensive oral motor therapy. Therefore, a comprehensive management strategy must be implemented throughout: the early stage focuses on life support and correction of malformations; mid-term interventions should aim to preserve auditory, visual, and speech functions; and the long term requires attention to hormone replacement therapy and psychological rehabilitation (3, 37). Although curative treatments such as rectifying downstream gene expression defects caused by CHD7 deficiency are currently still limited to animal studies (38), the aforementioned comprehensive management based on multidisciplinary collaboration is undoubtedly the cornerstone for improving the prognosis of patients with CHARGE syndrome.

Therefore, the diagnosis of CHARGE syndrome should be considered and confirmed through CHD7 gene testing in patients suspected of having esophageal atresia, as indicated by prenatal ultrasound, especially when accompanied by multiple malformations post-birth, and difficulties in discontinuing oxygen therapy and feeding challenges.

## Data Availability

The dataset presented in this study is available in a public repository. The repository name and accession number(s) can be found in the article/supplementary materials.
