# Supplementary material for: Case Report: Unveiling CHARGE syndrome: a neonatal case study with esophageal atresia and feeding difficulties
Source: Front Pediatr. 2025 Oct 23;13:1618512. doi: 10.3389/fped.2025.1618512 (PMC12588899; doi:10.3389/fped.2025.1618512)
Supplement: Supplementary file 1 [file Table1.docx]

Supplementary Material

## 1.Supplementary Figures


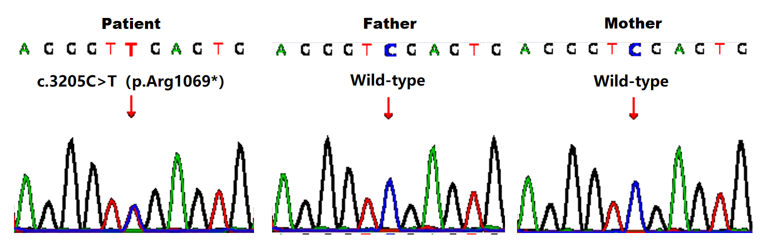


**Supplementary Figure S1.**

Sanger sequencing results. Genetic analysis showing the presence of a nonsense mutation of CHD7 gene in the infant (c.3205C>T, p.Arg1069* ) and the absence of mutation in the parents. The mutation location was marked by the arrows.

2.Supplementary Tables

Supplementary Table S1 Clinical diagnostic criteria for CHARGE syndrome

|  | **1998 Blake et al(17)** | **2005 Verloes(18)** | **2016 Hale et al(19)** |
| --- | --- | --- | --- |
| Major Criterion | - Coloboma - Choanal atresia - Characteristic ear abnormalities - Cranial nerve dysfunction | - Coloboma - Atresia of Choanae - Hypoplastic semi-circular | - Coloboma - Choanal atresia or cleft palate - Abnormal external, middle or inner ears - Pathogenic variants in the CHD7 gene |
| Minor Criterion | - Genital hypoplasia - Developmental delay - Cardiovascular malformations - Growth deficiency - Orofacial cleft - Tracheoesophageal-fistula - Distinctive face | - Rhombencephalic dysfunction - Hypothalamo-hypophyseal dysfunction - Abnormal middle or external ear - Malformation of mediastinal organs - Mental retardation | - Cranial nerve dysfunction (including hearing impairment) - Dysphagia or feeding difficulties - Abnormal brain structure - Developmental delay/autism or intellectual disability - Hypothalamic-pituitary dysfunction (deficiency in sex hormones/growth hormone) and gonadal abnormalities - Cardiac, renal anomalies, skeletal or digital abnormalities - Esophageal malformations |
| Diagnostic Criteria | - 4 major or - 3/4 major + 3/7 minor | - Typical CHARGE: 3 majors or 2/3 major + 2/5 minor - Partial/incomplete CHARGE: 2/3 major + 1/5 minor - Atypical CHARGE: 2/3 major or 1/3 major + 3/5 minors | - 2/4 major + 1/7 minor |

**Supplementary Table S2 published cases of CHARGE syndrome with EA/TEF**

| **Case** | **Author/year** | **Prenatal findings** | **EA/TEF type** | **Associated malformations** | **CHD7 variant** | **Interventions** | **Early outcomes** |
| --- | --- | --- | --- | --- | --- | --- | --- |
| 1(21) | Squires et al. / 1998 | Dandy-Walker cyst, Polyhydramnios | Gross C | Optic nerve coloboma, Heart disease,Micropenis, Undescended testes | Not mentioned | Surgical correction of TEF/EA, Management of hypocalcemia | Severe growth failure, developmental delay |
| 2(32) | Farquhar J et al. / 2002 (Twin 1) | \| Polyhydramnios \| \| --- \| | Gross C | Bilateral microphthalmos, Extensive retinal colobomata, Patent ductus arteriosus (PDA), Aberrant right brachiocephalic artery, Bilateral grade III intraventricular haemorrhage (IVH), | Not mentioned | Surgical repair of EA/TEF, Conservative management of IVH and hydrocephalus | Severe developmental delay and growth problems |
| 3(32) | Carachi et al. / 2002 (Twin 2) | \| Polyhydramnios \| \| --- \| | Gross C | Right microphthalmos, Large optic disc coloboma, Patent ductus arteriosus (PDA),Sensorineural deafness | Not mentioned | Surgical repair of EA/TEF | Severe developmental delay and growth problems |
| 4(30) | Jongmans MC et al./2006(patient 4) | not mentioned | not mentioned | Heart defect,External ear anomaly,Hearing loss,Vestibular dysfunction ,Cleft lip， Cleft palate | c.1388delG （p.Gly463fs） | Surgical repair of EA/TEF | Survival, not specifically mentioned |
| 5(30) | Jongmans MC et al./2006(patient 21) | Not mentioned | Not mentioned | Microphthalmia,Heart defect,Atresia of choanae,External ear anomaly, Hearing loss, Facial nerve palsy | c.5405-17G＞A | Surgical repair of EA/TEF | Survival, not specifically mentioned |
| 6(30) | Jongmans MC et al./2006(patient 22) | Not mentioned | Not mentioned | Heart Defect,atresia of choanae,External ear anomaly,Hearing loss, Vestibular dysfunction | c.5418C＞G(P.Asn1807X 22q11del) | Surgical repair of EA/TEF | Survival, not specifically mentioned |
| 7(30) | Jongmans MC et al./2006(patient 25) | Not mentioned | Not mentioned | Coloboma，Heart defect,Micropenis/ cryptorchidism, External ear anomaly, Hearing loss, Vestibular dysfunction, Facial nerve palsy | c.5680_5681delAG(p.Ser1894fs) | Not mentioned | Deceased |
| 8(30) | Jongmans MC et al./2006(patient 27) | Not mentioned | Not mentioned | Heart Defect,atresia of choanae,External ear anomaly | c.5752_5753dupA(P.Thr1918fs) | Surgical repair of EA/TEF | Survival, not specifically mentioned |
| 9(30) | Jongmans MC et al./2006(patient 30) | Not mentioned | Not mentioned | Coloboma,Micropenis/ cryptorchidism,External ear anomaly,Hearing loss | c.5982G＞A(p.Trp1994X) | Surgical repair of EA/TEF | Survival, not specifically mentioned |
| 10(30) | Jongmans MC et al./2006(patient 31) | Not mentioned | Not mentioned | Coloboma,External ear anomaly,Hearing loss,Vestibular dysfunction,Cleft lip,Cleft palate | c.6051T＞A (p.Cys2017X) | Not mentioned | Deceased |
| 11(30) | Jongmans MC et al./2006(patient 33) | Not mentioned | Not mentioned | Coloboma,Microphthalmia,Atresia of choanae,External ear anomaly,Hearing loss | c.6079C＞T (p.Arg2027X) | Not mentioned | Deceased |
| 12(28) | Janda et al. / 2007 | Polyhydramnios | Not mentioned | Bilateral choanal atresia, Retinal coloboma, **Cup-shaped ears**, Micropenis.Heart defects | Not mentioned | Correction of EA/TEF, PDA resection, choanal dilatation, fundoplication | Clinically well |
| 13(31) | Delahaye et al. / 2007 | Polyhydramnios (from 26 weeks) | Gross C | Bilateral cleft lip/palate, Complex heart defect,Left cryptorchidism, Malacia of right main bronchus | c.2501C>T (p.Ser834Phe) | Surgical repair of EA/TEF and cleft lip/palate | Survived. Significant developmental delay |
| 14(29) | Lee et al. / 2008 | Mild polyhydramnios | Gross C | Choanal atresia, Hearing loss, Coloboma, Genital hypoplasia, Growth retardation. | Not mentioned | Gastrotomy , Surgical repair of EA/TEF | Severe post-operative course |
| 15(35) | Upadhyaya V.D. et al. /(2012) | Not mentioned | Gross C | Unilateral absence of the external ear (pinna) | Not mentioned | surgery repair | Uneventful postoperative course. Patient was well at 2-year follow-up |
| 16(33) | Chang et al. / 2014 | not mentioned | Gross A | Bilateral iris and chorioretinal colobomata, Left microphthalmia, Heart defects,Dysmorphic face | not mentioned | Gastrostomy and colostomy | Survived neonatal period |
| 17(34) | Cappuccio et al. / 2014(Patient 1) | Not mentioned | Gross C | Left optic nerve coloboma, Sensorineural hearing loss, **Cleft lip/palate** | c.4353+3A>G (p.Ala1396_Gly1451del) | Not mentioned | Psychomotor delay |
| 18(22) | Yokota et al. / 2022 | Right lip and palate cleft, Anomalous origin of the right subclavian artery, Absence of the right kidney, Mega cisterna magna, Cerebellar hypoplasia | Gross E | Heart defects, Cleft lip/palate , Retarded growth/development,Ear anomalies/Sensorineural deafness. | Large deletion of exons 14-15 | Surgical repai of H-type TEF via cervical approach with sternohyoid muscle flap interposition | Respiratory failure,Feeding difficulties, Neurodevelopmental delay |
| 19(36) | Khattar et al. / 2023 (patient 2) | not mentioned | Gross C | Bilateral choanal atresia, right eye coloboma, micropenis | c.1480C>T (p.Arg494*) | Surgical repair, G-tube, respiratory support | Survival, require tube feeding |
| 20(36) | Khattar et al. / 2023 (patient 8) | not mentioned | Gross C | Aberrant right subclavian artery, VSD, right coloboma, tracheobronchomalacia, rib synostosis | 4:c.4393C>T (p.Arg1465*) | Surgical repair, G-tube, respiratory support | Survival,require tube feeding |
| 21(36) | Khattar et al. / 2023 (patient 9) | not mentioned | Gross C | AV canal defect, thymic hypoplasia, bicuspid aortic valve, right aortic arch, bilateral choanal atresia, horseshoe kidney, ectrodactyly, bilateral colobomas, club foot | 4:c.4480C>T (p.Arg1494*) | Surgical repair, G-tube, respiratory support | Survival,require tube feeding |
